# Supplementary figures and images for: Activation of autophagy reverses progressive and deleterious protein aggregation in PRPF31 patient‐induced pluripotent stem cell‐derived retinal pigment epithelium cells
Source: Clin Transl Med. 2022 Mar 16;12(3):e759. doi: 10.1002/ctm2.759 (PMC8926896; doi:10.1002/ctm2.759)

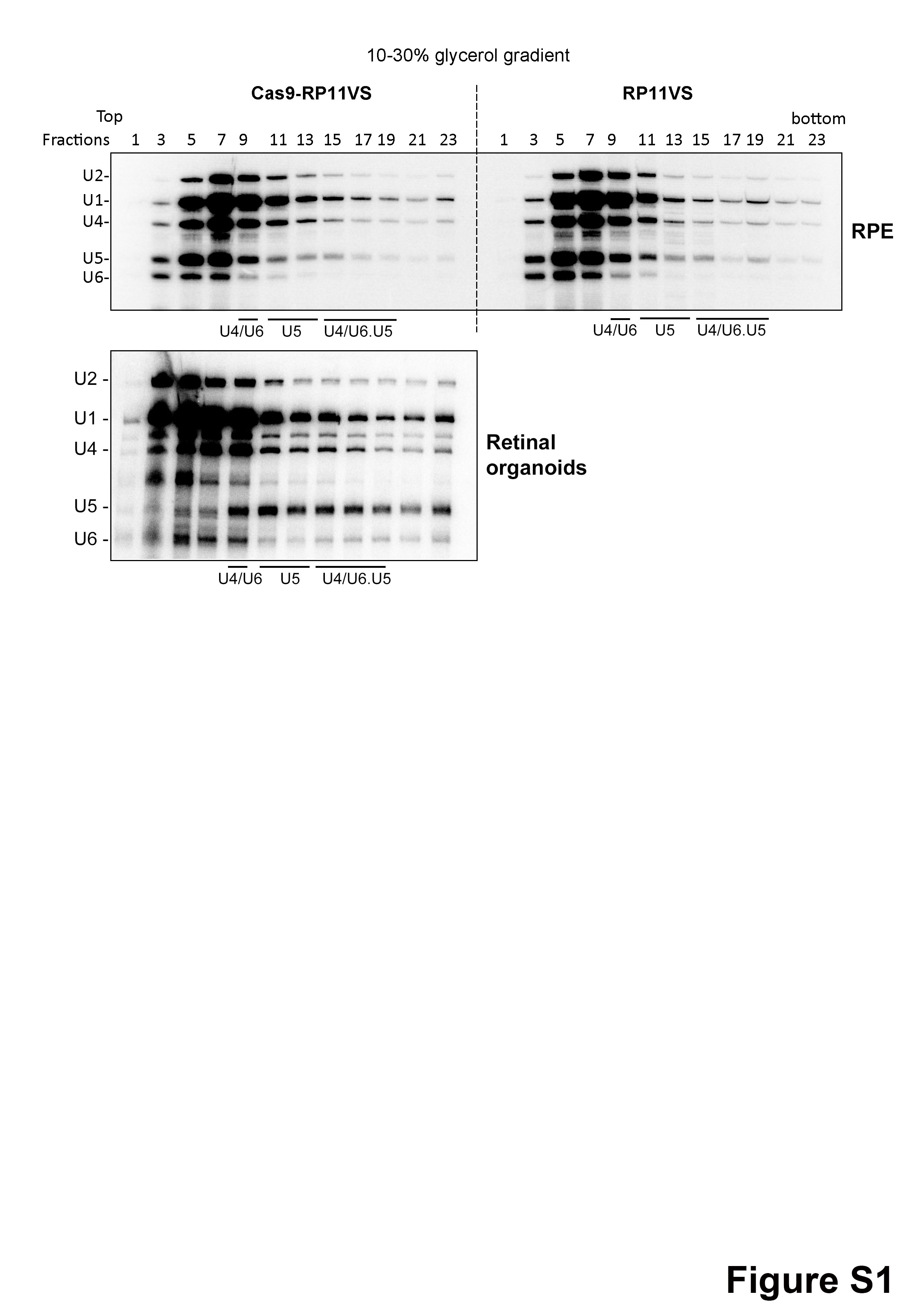

Supplement: Supplementary file 1 — Supporting Information [file CTM2-12-e759-s001.jpg]

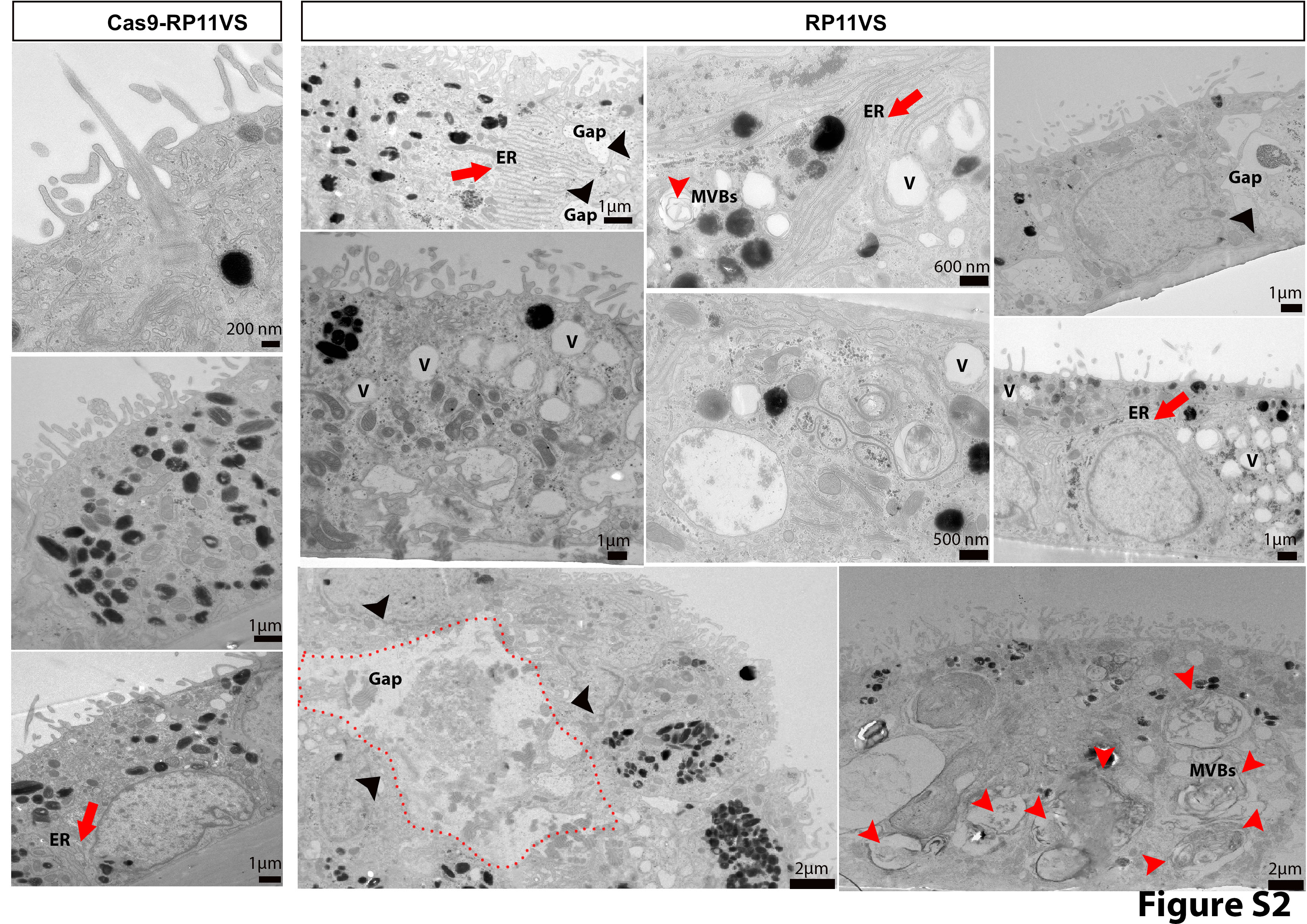

Supplement: Supplementary file 2 — Supporting Information [file CTM2-12-e759-s003.jpg]

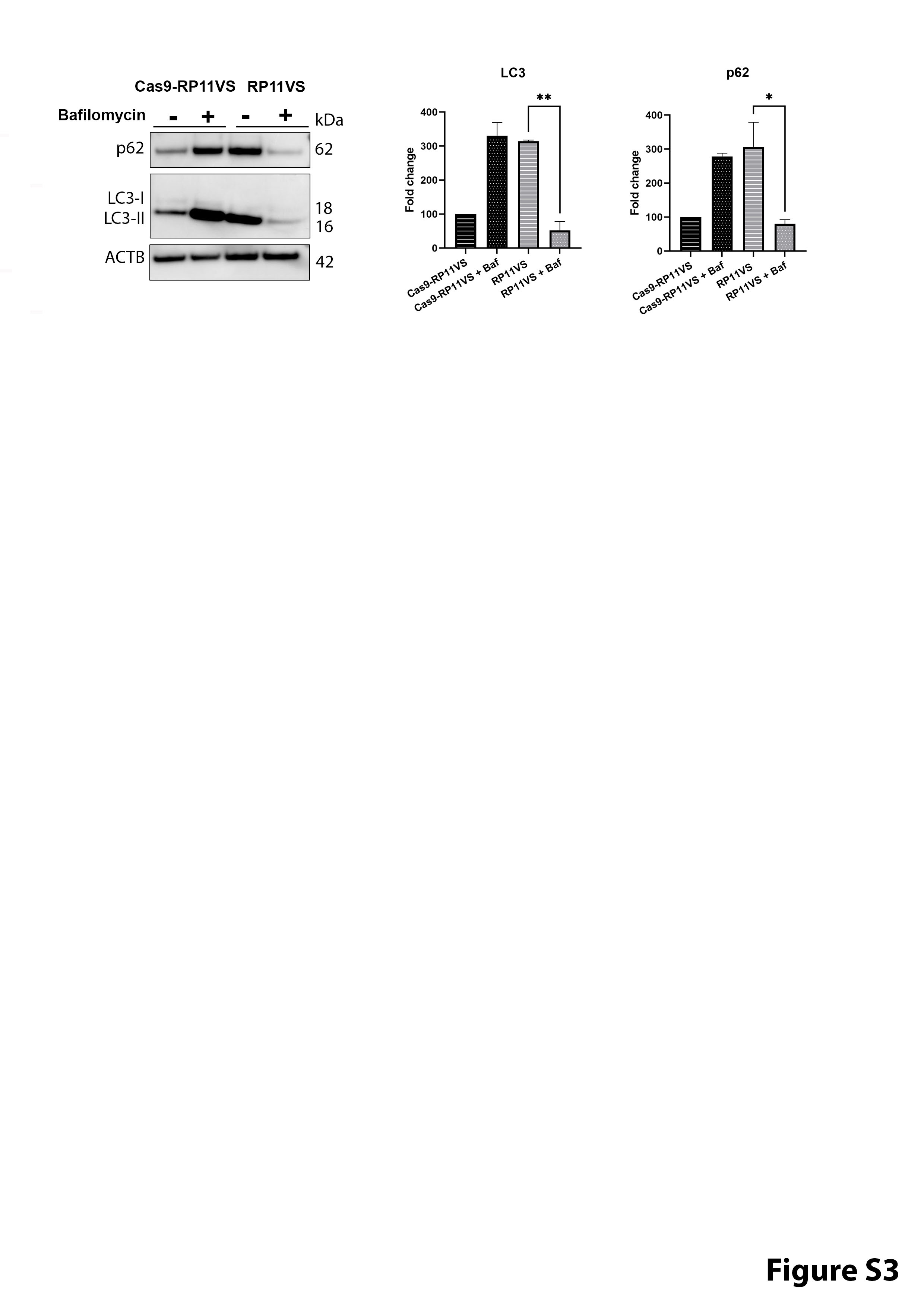

Supplement: Supplementary file 3 — Supporting Information [file CTM2-12-e759-s009.jpg]

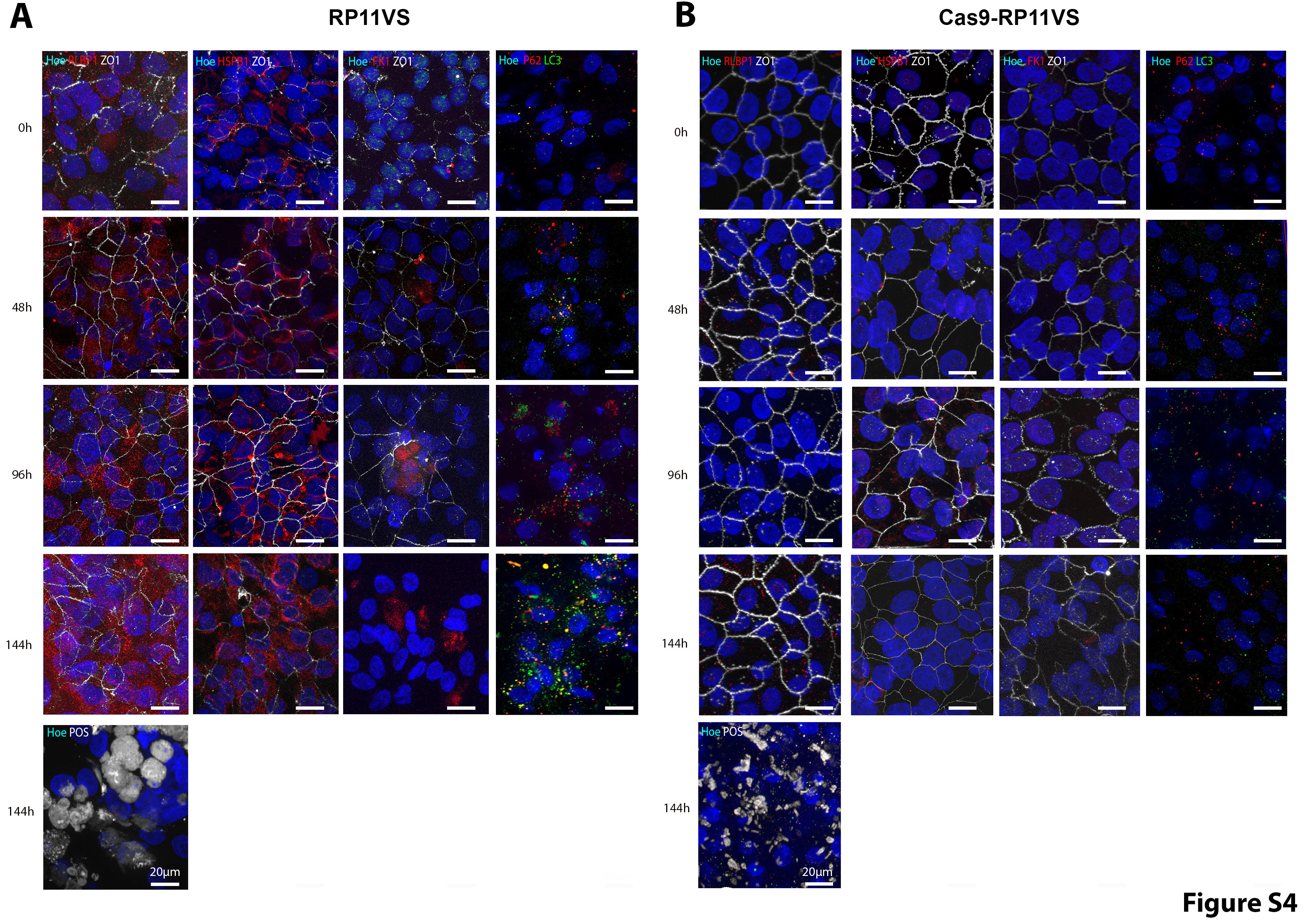

Supplement: Supplementary file 4 — Supporting Information [file CTM2-12-e759-s004.jpg]

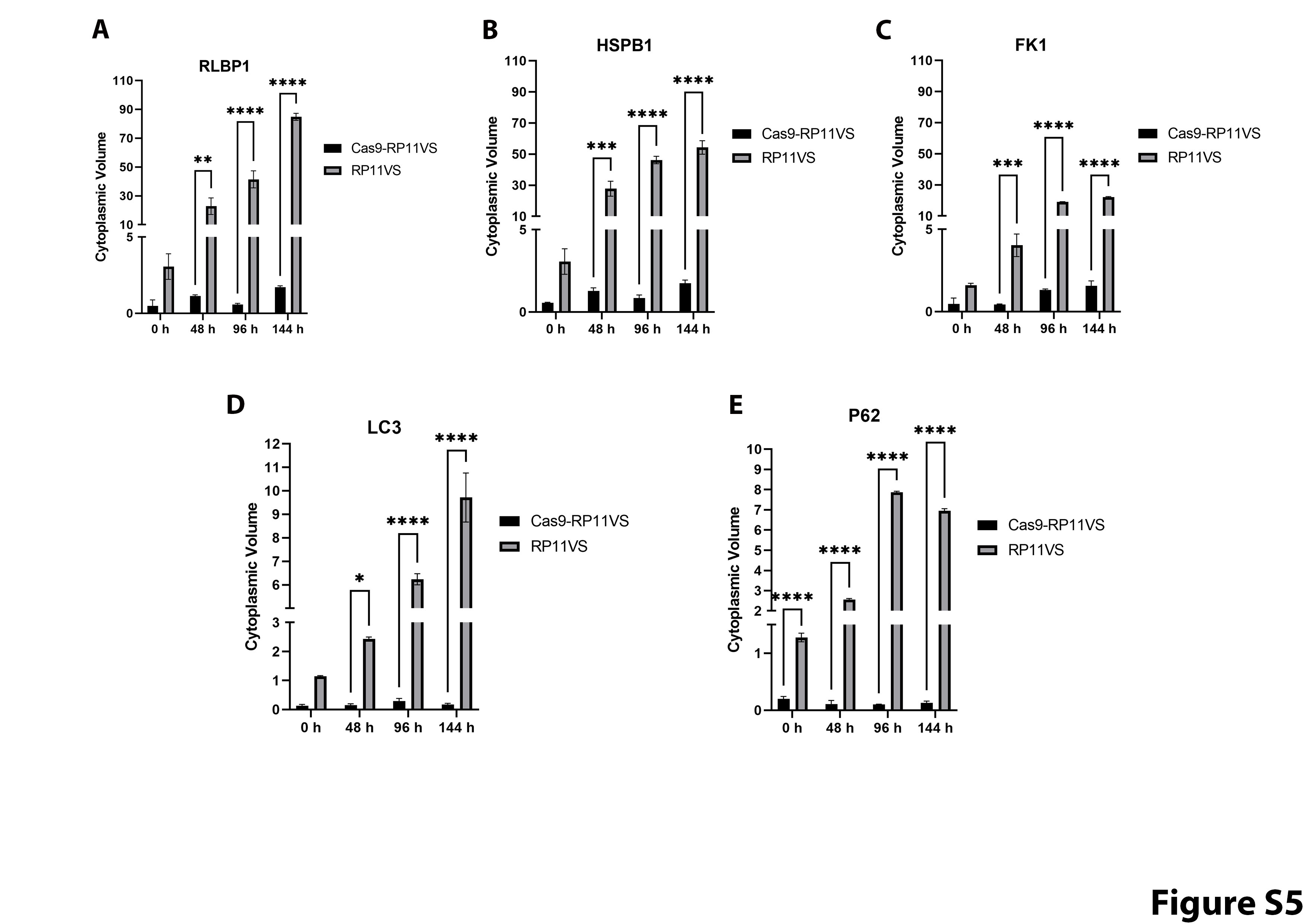

Supplement: Supplementary file 5 — Supporting Information [file CTM2-12-e759-s012.jpg]

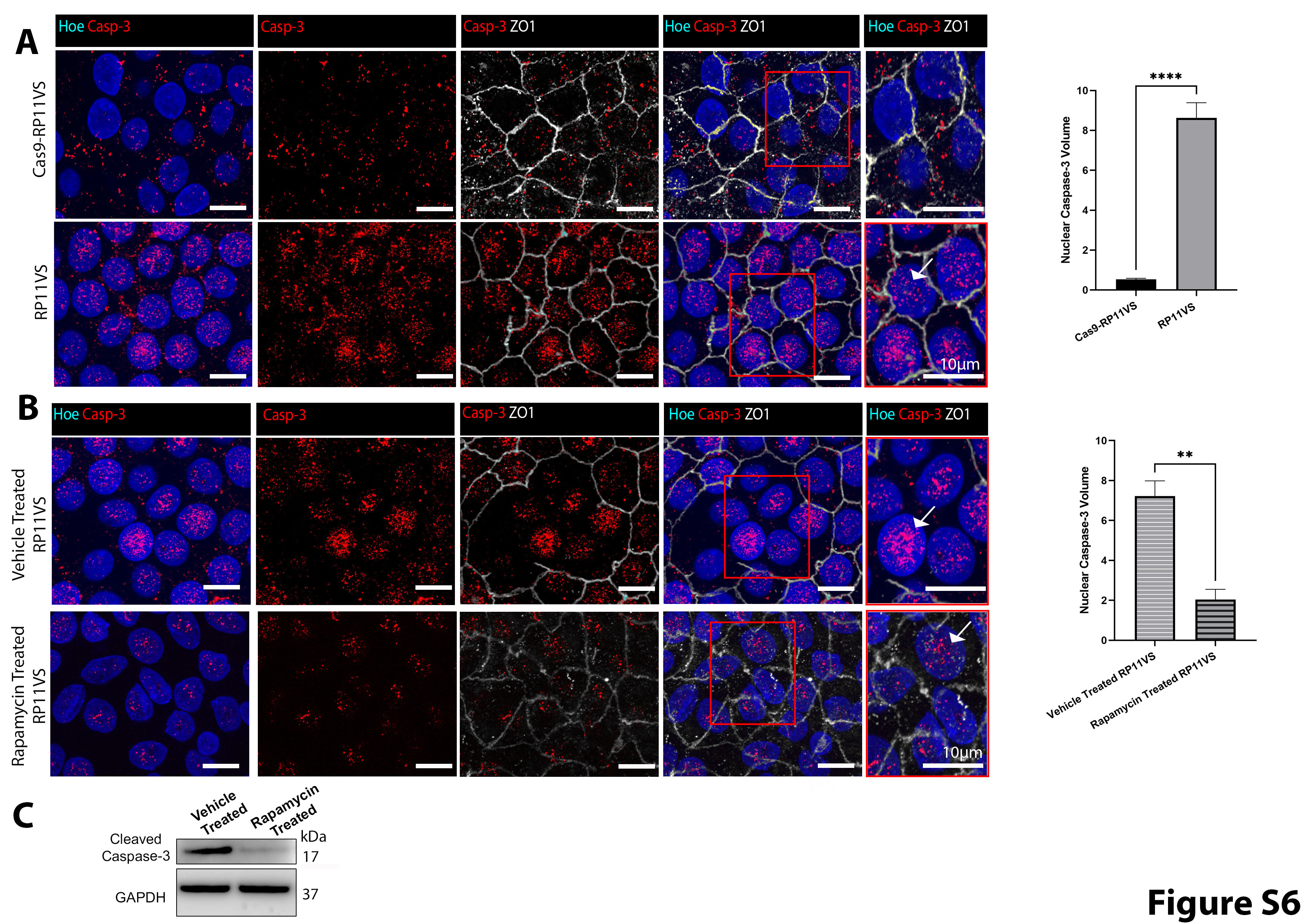

Supplement: Supplementary file 6 — Supporting Information [file CTM2-12-e759-s011.jpg]

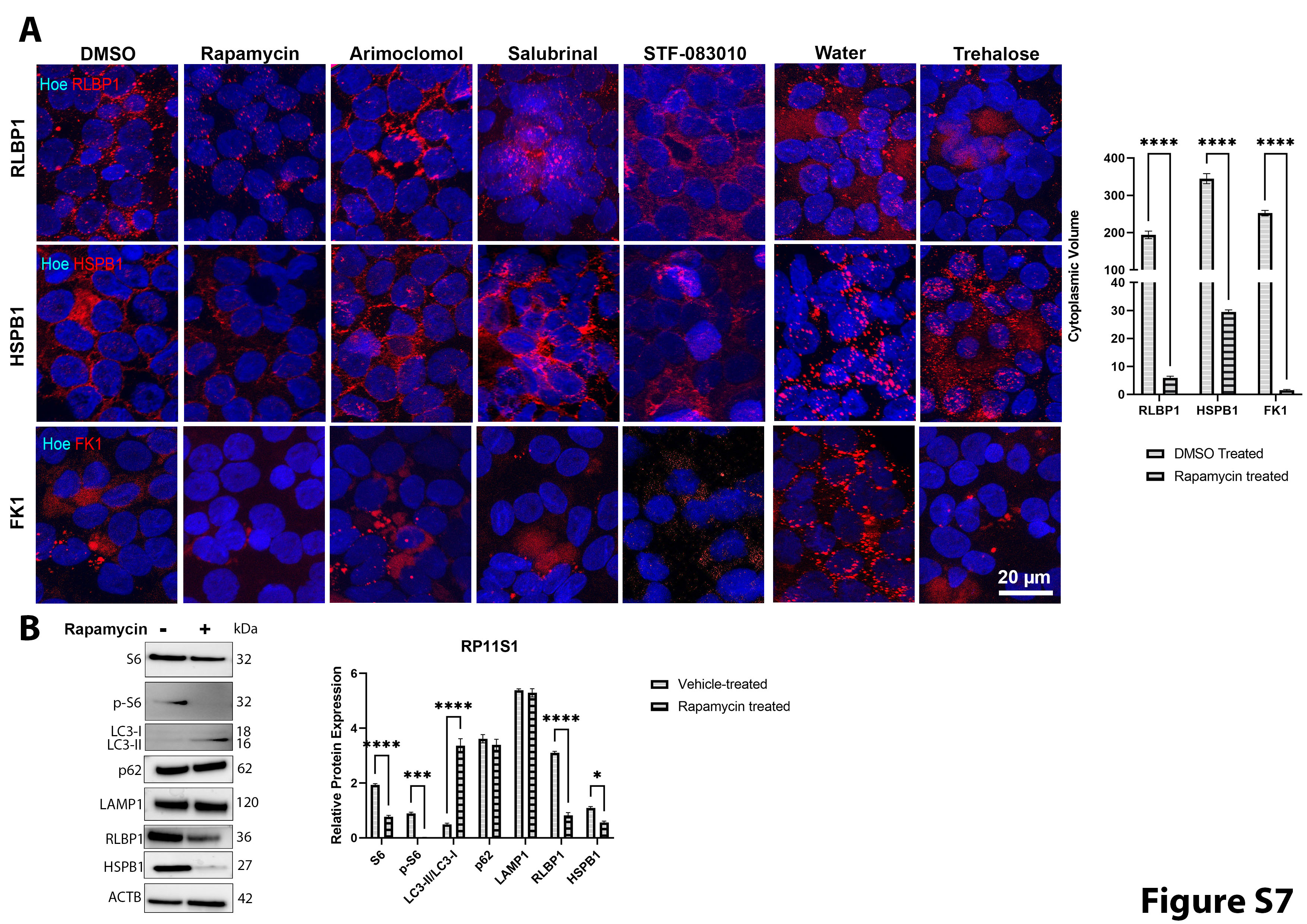

Supplement: Supplementary file 7 — Supporting Information [file CTM2-12-e759-s006.jpg]

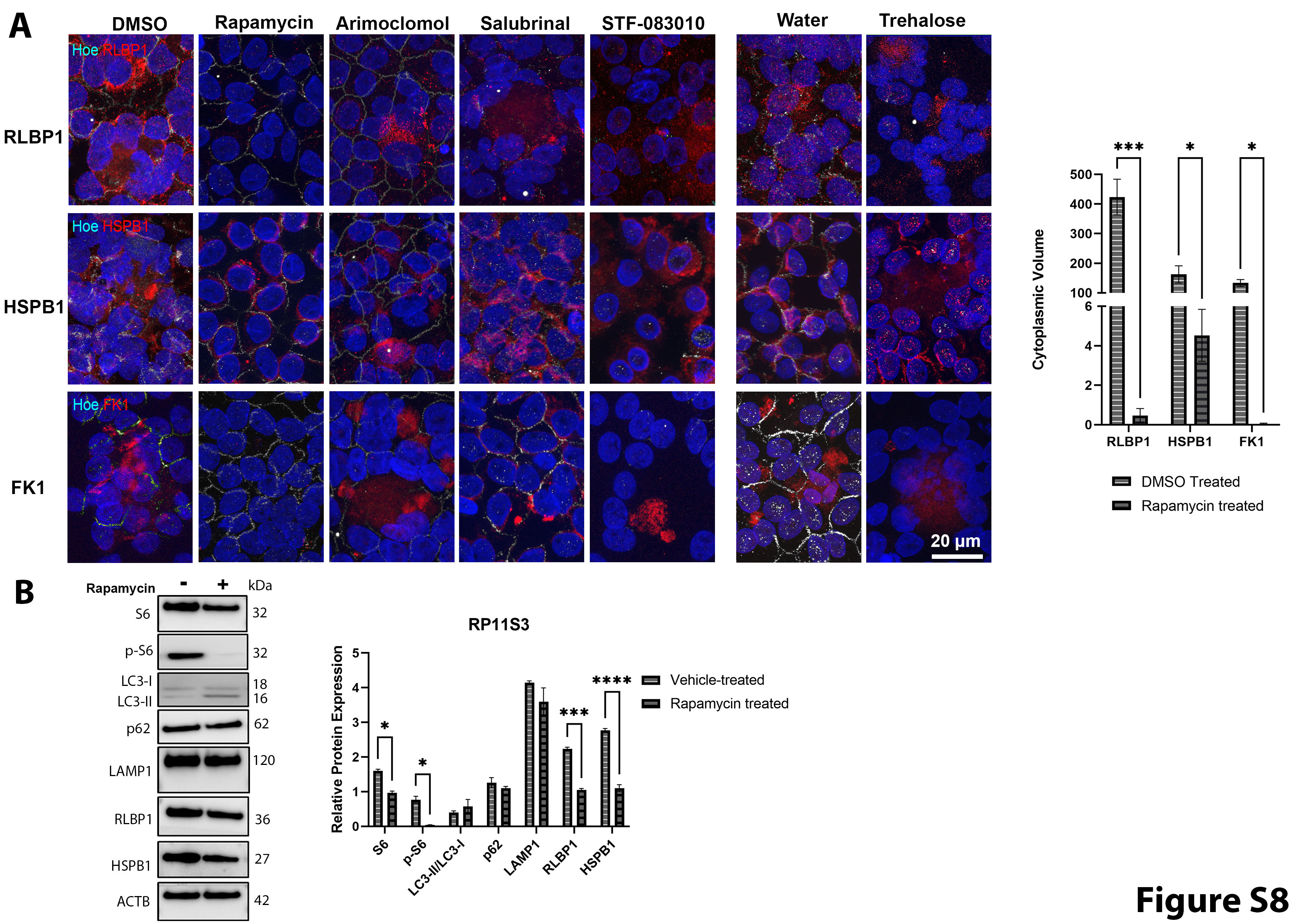

Supplement: Supplementary file 8 — Supporting Information [file CTM2-12-e759-s010.jpg]
